# 气相色谱-质谱法同时测定化妆品中18种氯代烃类有机溶剂

**DOI:** 10.3724/SP.J.1123.2020.05010

**Published:** 2021-03-08

**Authors:** Juan TANG, Xiaoqing FEI, Jia ZHOU, Kai QIAN, Shaowei DONG, Lihua CAO, Youchao DING

**Affiliations:** 1.南京海关工业产品检测中心, 江苏 南京 210019; 1. Industrial Products Testing Center, Nanjing Customs, Nanjing 210019, China; 2.南京海关动植物与食品检测中心, 江苏 南京 210019; 2. Animal, Plant and Food Inspection Center, Nanjing Customs, Nanjing 210019, China; 3.南京金检检验有限公司, 江苏 南京 210019; 3. Nanjing Jinjian Inspection Co., Ltd, Nanjing 210019, China

**Keywords:** 气相色谱-质谱, 氯代烃类有机溶剂, 化妆品, gas chromatography-mass spectrometry (GC-MS), chlorinated hydrocarbon organic solvents, cosmetics

## Abstract

建立了同时测定化妆品中18种氯代烃类有机溶剂的气相色谱-质谱(GC-MS)检测方法。样品在饱和氯化钠溶液中由正十四烷振荡提取后,以Agilent J&W DB-624超高惰性毛细管柱(30 m×0.25 mm×1.4 μm)为分离色谱柱进行分析,以电子轰击(EI)源、SIM模式进行质谱监测,外标法定量。结果表明,18种化合物在19 min内完成色谱分离分析,检出限(LOD, *S/N*=3)和定量限(LOQ, *S/N*=10)分别为0.033~0.049 mg/L和0.10~0.15 mg/L, 18种氯代烃类有机溶剂在0.2~100 mg/L线性范围内线性关系良好,相关系数(*R*^2^)均不小于0.9992。以阴性样品口红(固体)和漱口水(液体)为样品基质,在不同添加水平下,18种氯代烃类有机溶剂的平均回收率分别为92.4%~103.1%和93.3%~102.4%,相对标准偏差(RSD, *n*=6)分别为3.1%~5.3%和2.8%~5.4%。采用该方法对115个化妆品样品进行测定,3个指甲油样品均检测出四氯乙烯,测定值为11.4~42.0 g/kg。研究建立的方法采用高沸点溶剂作为进样溶剂,取消溶剂延迟时间,使只能在溶剂延迟时间出峰的化合物得到有效色谱分离,分析时间短,且重复性好,灵敏度高,可同时检测各种化妆品中多种氯代烃类有机溶剂。该方法的建立为我国化妆品中氯代烃类有机溶剂检测标准的制订和质量安全监控提供了参考。

## Abstract (en)

Organic solvents can be used to dissolve and disperse flavors, bactericides, preservatives, surfactants, oils, and coloring agents during the production of cosmetics. However, harmful chlorinated hydrocarbon organic solvents are found in cosmetics such as manicure products, anti-acne products, and perfumes. Long-term contact with such cosmetics will have an adverse effect on the consumers’ health. Past research has focused on very few chlorinated hydrocarbon organic solvents in cosmetics. Most organic solvents with low boiling points are typically determined by headspace-gas chromatography-mass spectrometry. In this study, a high-boiling-point solvent was used as the injection solvent, and the solvent delay time was cancelled. The compounds that could only peak during the solvent delay time were effectively separated. A method coupling sample pretreatment with gas chromatography-mass spectrometry (GC-MS) was developed for the simultaneous determination of 18 chlorinated hydrocarbon organic solvents in cosmetics: vinylidene chloride, dichloromethane, *trans*-1,2-dichloroethylene, 1,1-dichloroethane, *cis*-1,2-dichloroethylene, chloroform, 1,1,1-trichloroethane, carbon tetrachloride, 1,2-dichloroethane, trichloroethylene, 1,1,2-trichloroethane, tetrachloroethylene, 1,1,1,2-tetrachloroethane, 1,1,2,2-tetrachloroethane, 1,2,3-trichloropropane, pentachloroethane, hexachloroethane, and hexachloro-1,3-butadiene. These 18 solvents have a wide range of polarities and a notable difference in volatilities, in addition to many isomers and structural analogs, which renders their separation difficult. Therefore, the separation effect of three kinds of GC columns with different polarities was compared. *n*-Tetradecane, an injection solvent with good solubility, was selected as the extraction solvent. An organic solvent with high polarity has low extraction rate because of its weak polarity. Adding sodium chloride solution to the sample to induce the “salting out” effect could change the partition coefficient of the components, thereby improving the extraction rate. Therefore, the concentration of the sodium chloride solution added to the sample was optimized. In this work, liquid-liquid extraction was the main extraction process, so the effects of different shaking times, temperatures, and frequencies on the extraction rate were discussed. The optimized results are as follows: at normal temperature, the sample dispersed or dissolved in saturated sodium chloride solution was extracted by *n*-tetradecane at an oscillating speed of 100 r/min for 20 min. Separation was performed on an Agilent J&W DB-624 column (30 m×0.25 mm×1.4 μm) by GC-MS with an electrospray ionization (EI) source in the selected ion monitoring (SIM) mode. The external standard method was used for quantitative determination. The 18 compounds could be analyzed within 19 min. The linear equations, linear correlation coefficients, and linear ranges were obtained by analyzing a series of mixed standard working solutions. The limits of detection (LODs, *S/N*=3) and limits of quantification (LOQs, *S/N*=10) of the 18 components were determined. The negative lipstick (solid) and mouthwash (liquid) samples were used as the spiked sample matrix at three levels, and the recoveries and precisions were calculated. The calibration curves showed good linearities for the 18 chlorinated hydrocarbon organic solvents in range of 0.2-100 mg/L, with correlation coefficients (*R*^2^) not less than 0.9992. The LODs and LOQs were in the range of 0.033-0.049 mg/L and 0.10-0.15 mg/L, respectively. The average recoveries of the 18 chlorinated hydrocarbon organic solvents in lipstick (solid) and mouthwash (liquid) were 92.4%-103.1% and 93.3%-102.4% respectively; the corresponding relative standard deviations (RSDs) were 3.1%-5.3% and 2.8%-5.4% (*n*=6). This method was used to determine 115 different types of cosmetics, and tetrachloroethylene was detected in three nail polishes. With its advantages of high sensitivity, good precision, and accuracy, the developed method is suitable for the quantitative analysis of the aforesaid 18 compounds in all kinds of cosmetics. The study findings would serve as a reference for the quality and safety monitoring of cosmetics.

有机溶剂在化妆品生产过程中可用于溶解和分散香精、杀菌防腐剂、表面活性剂、油脂和着色剂等,但在美甲用品、防粉刺化妆品和香水等产品中时有发现一些有毒有害氯代烃类有机溶剂的存在。长时间接触含有此类氯代烃类有机溶剂的化妆品会损害消费者的身体健康,如长期接触三氯乙烯和二氯甲烷会引起中毒性神经衰弱、植物神经紊乱和中毒性神经末梢炎,长期接触氯仿、四氯化碳、三氯乙烯、四氯乙烯和三氯丙烷等会引起脂肪肝、肝细胞坏死和肾功能减退等。《化妆品安全技术规范》(2015版)中明确规定了1,1,2-三氯乙烷、1,2,3-三氯丙烷、氯仿、四氯化碳、二氯乙烷类、二氯乙烯类、四氯乙烯、二氯甲烷等氯代烃类有机溶剂禁止或限用于化妆品的生产。因此,建立一个快速且有效的化妆品中氯代烃类有机溶剂的检测方法变得十分迫切。

目前,国内外有关氯代烃类有机溶剂检测的文献报道中,检测基质主要集中于药物^[1,2,3,4,5]^、食品^[6,7]^、纺织品^[8]^、塑料制品^[9,10]^、皮革^[11,12,13]^、染整助剂^[14]^和化妆品^[15,16,17,18]^等,检测方法主要有气相色谱法(GC)^[19,20]^和气相色谱-质谱法(GC-MS)^[21,22]^。已报道文献中,涉及的氯代烃类有机溶剂种类较少。GC-MS结合了气相色谱优良的分离性和质谱鉴定的高选择性,测定挥发性氯代烃类有机溶剂具有较高的灵敏度和准确度。对于沸点较低的氯代烃类有机溶剂,多数以顶空-GC-MS测定,但本文不采用顶空进样,使用高沸点试剂作为进样溶剂,取消溶剂延迟时间,建立了GC-MS测定化妆品中18种氯代烃类有机溶剂的方法。

## 1 实验部分

### 1.1 仪器、试剂和材料

Trace DSQⅡ气相色谱-质谱仪(美国Thermo Fisher公司); LDZ5-2离心机(美国Sigma公司); Milli-Q超纯水系统(美国Millipore公司); HY-4A调速多用振荡器(常州润华电器有限公司); PL602-L和ML54型电子天平(感量0.01 g和0.0001 g,梅特勒-托利多仪器上海有限公司);聚四氟乙烯有机相针式滤器(13 mm×0.45 μm,南京泰谱瑞仪器设备有限公司)。

标准品:二氯甲烷、三氯甲烷、四氯化碳、1,1-二氯乙烷、1,2-二氯乙烷、1,1,1-三氯乙烷、1,1,2-三氯乙烷、1,1,1,2-四氯乙烷、1,1,2,2-四氯乙烷、五氯乙烷、六氯乙烷、1,2,3-三氯丙烷、1,1-二氯乙烯、顺-1,2-二氯乙烯、反-1,2-二氯乙烯、三氯乙烯、四氯乙烯和六氯丁二烯均购于德国Dr. Ehrenstorfer公司,纯度≥99.0%。正十四烷(纯度大于99%, GC级,上海安谱实验技术股份有限公司);氯化钠和无水硫酸钠(分析纯,南京化学试剂股份有限公司)。

115个实际化妆品样品均购于市场。

### 1.2 标准溶液的配制

标准储备液:准确称取18种氯代烃类有机溶剂各100 mg,分别用正十四烷溶解并定容至10 mL,配制成质量浓度均为10 g/L的18种氯代烃类有机溶剂标准储备液,于4 ℃冷藏保存。

标准工作液:分别移取0.5 mL上述18种标准储备液,置于10 mL容量瓶中,并用正十四烷定容至刻度,配制成500 mg/L的混合标准工作液;用正十四烷逐级稀释,配制成质量浓度分别为0.1、0.2、0.5、1.0、2.0、5.0、10.0、20.0、50.0、100.0 mg/L的系列混合标准工作液。

### 1.3 样品前处理

称取1.0 g(精确至0.01 g)样品,置于50 mL离心管中,加入5 mL饱和氯化钠溶液超声分散后,加入5 mL正十四烷,常温下以100 r/min振荡提取20 min,然后以8000 r/min离心3 min后,取部分上清液,过膜后供GC-MS测定。必要时用正十四烷稀释后再进样分析。

### 1.4 分析条件

色谱条件:色谱柱为Agilent J&W DB-624超高惰性毛细管柱(30 m×0.25 mm×1.4 μm);进样口温度为250 ℃;载气为高纯氦气(99.999%);流速为1.0 mL/min;分流进样,分流比为10∶1。升温程序为初始温度50 ℃,保持5 min,以10 ℃/min的速率升温至240 ℃,保持2 min。进样量为1.0 μL。

质谱条件:电子轰击(EI)离子源,电离能量为70 eV,离子源温度为230 ℃,四级杆温度为150 ℃,传输线温度为280 ℃,溶剂延迟时间为0 min, 19 min关闭检测器,选择离子监测(SIM)模式,18种氯代烃类有机溶剂的质谱分析参数见表1。

**表1:** 18种氯代烃类有机溶剂的保留时间和监测离子

No.	Compound	Retention time/min	Monitoring ions (*m/z*)
1	vinylidene chloride	2.85	61^*^, 96, 63
2	dichloromethane	3.34	49^*^, 84, 86
3	*trans*-1,2-dichloroethylene	3.68	61^*^, 96, 98
4	1,1-dichloroethane	4.22	63^*^, 65, 83
5	*cis*-1,2-dichloroethylene	5.13	61^*^, 96, 98
6	chloroform	5.66	83^*^, 85, 4 7
7	1,1,1-trichloroethane	5.96	97^*^, 99, 61
8	carbon tetrachloride	6.25	117^*^, 119, 82
9	1,2-dichloroethane	6.57	62^*^, 64, 49
10	trichloroethylene	7.59	130^*^, 132, 95
11	1,1,2-trichloroethane	10.18	97^*^, 83, 61
12	tetrachloroethylene	10.40	166^*^, 129, 94
13	1,1,1,2-tetrachloroethane	11.81	131^*^, 117, 95
14	1,1,2,2-tetrachloroethane	13.66	83^*^, 95, 60
15	1,2,3-trichloropropane	13.71	75^*^, 110, 61
16	pentachloroethane	14.60	167^*^, 117, 130
17	hexachloroethane	16.14	117^*^, 201, 166
18	hexachloro-1,3-butadiene	18.45	225^*^, 190, 260

* Quantitative ion.

## 2 结果与讨论

### 2.1 色谱柱的选择

本研究涉及的18种氯代烃类有机溶剂极性跨度大,挥发性差别大,包括多种同分异构体和结构相似物,分离难度较大。实验分别比较了Agilent DB-1 (30 m×0.25 mm×0.25 μm)、DB-5(30 m×0.25 mm×0.25 μm)、DB-624(30 m×0.25 mm×1.4 μm)、DB-WAX(30 m×0.25 mm×0.25 μm)等不同极性的毛细管柱对18种氯代烃类有机溶剂色谱分离效果的影响。结果可知,18种氯代烃类有机溶剂在DB-624毛细管柱上均有较高的响应,在DB-1和DB-5毛细管柱上,待测组分虽有良好的分离,但响应值相对较低。DB-WAX毛细管柱极性较强,在高温条件下,柱流失较严重。综上,本文选择DB-624毛细管色谱柱作为分析柱,它由6%的氰丙基-苯基和94%的二甲基聚硅氧烷组成,属于中等极性柱,对非极性和极性化合物均具有较好的分离效果,1,1,2,2-四氯乙烷与1,2,3-三氯丙烷色谱峰虽未完全分离,但根据它们之间不同的碎片离子可分别进行定性和定量。在优化的色谱条件下,18种氯代烃类有机溶剂的SIM色谱图见图1。

**图1:**
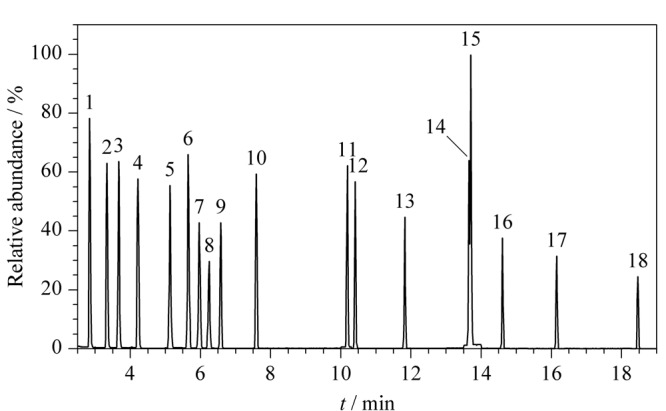
18种氯代烃类有机溶剂的SIM色谱图

### 2.2 提取溶剂的选择

18种氯代烃类有机溶剂易挥发,1,1-二氯乙烯和二氯甲烷等有机溶剂色谱保留时间短,如使用常规试剂作为提取溶剂,较早出峰的一些待测组分的色谱峰会与溶剂峰重合。因此,实验选择高沸点溶剂正十四烷作为溶剂,取消溶剂延迟时间,18种待测组分的色谱峰在正十四烷溶剂峰前完全出峰,且均有较高响应,保证了待测组分的有效色谱分离和准确定量,待测组分分离结束后关闭检测器,高温烘出溶剂和杂质,延缓了灯丝的使用寿命,同时有效降低了离子源污染。

### 2.3 提取溶液的优化

实验选用具有良好溶解性的高沸点溶剂正十四烷作为提取溶剂,但正十四烷极性较弱,直接将其作为提取溶剂,会导致极性较大的有机溶剂提取率相对较低。因此待测样品中加入氯化钠溶液以产生“盐析”效应,改变待测组分的分配系数以提高提取率。结果表明,质量浓度小于50 g/L的氯化钠溶液作用较低,饱和氯化钠溶液效果最为明显,“盐析”效应对极性待测组分的提取效率改善尤为明显。经过优化可知,1.0 g样品中加入5 mL饱和氯化钠溶液可达到明显效果,加标回收率和灵敏度均能达到检测要求。饱和氯化钠对18种氯代烃类有机溶剂提取率的影响见图2。

**图2:**
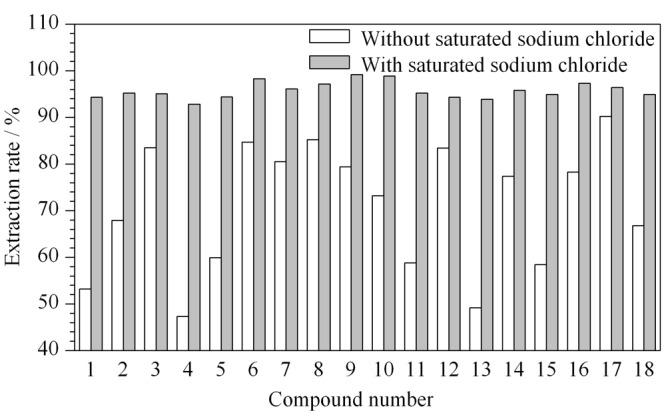
饱和氯化钠对18种氯代烃类有机溶剂提取率的影响

### 2.4 提取条件的优化

实验以液液萃取为主,因此振摇时间、振摇温度和振摇频率是提取率的关键因素。结果表明,升高振摇温度会导致极易挥发的有机溶剂挥发而使提取率下降,而振摇频率过快,会导致提取溶液乳化严重。经过优化,本文选择在常温下振摇,振摇频率为100 r/min。

实验讨论了不同振摇时间(0、5、10、15、20、25、30 min)对分析结果的影响,结果见图3。随着振摇时间的延长,18种氯代烃类有机溶剂的提取率也随之增加,当振摇时间为20 min时,提取率最高。因此,本文将振摇时间定为20 min。

**图3:**
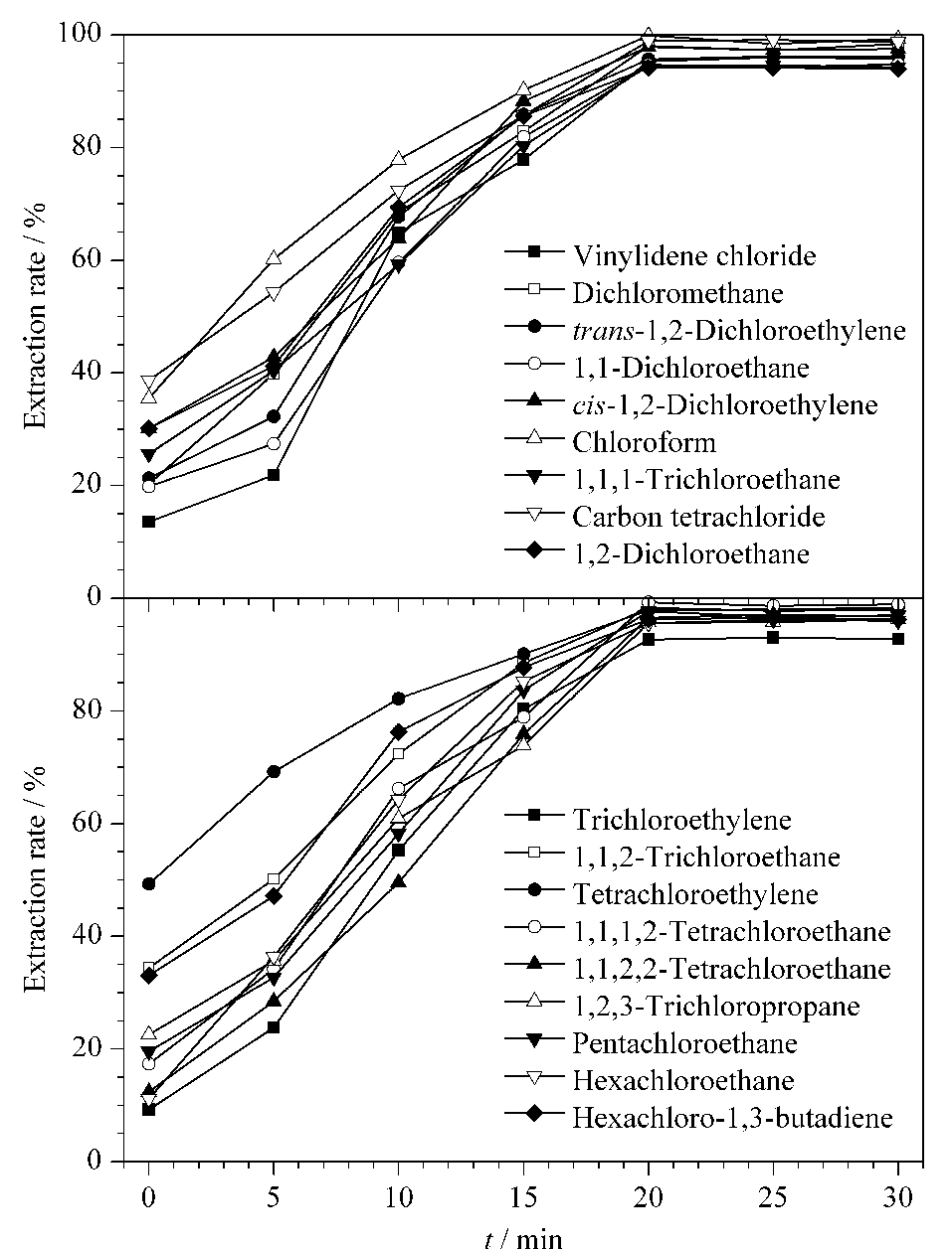
不同振摇时间对18种氯代烃类有机溶剂提取率的影响

### 2.5 方法学评价

2.5.1 线性范围、检出限和定量限

对1.2节中的系列混合标准工作液进行分析,以各组分定量离子的峰面积(*Y*)和对应的质量浓度(*X*, mg/L)进行线性回归,得到线性方程,线性相关系数(*R*^2^)均不小于0.9992。表明18种氯代烃类有机溶剂在0.2~100 mg/L内线性关系良好。

在阴性样品中分别添加不同水平的待测组分,根据3倍和10倍信噪比分别确定18种氯代烃类有机溶剂的检出限(LOD, *S/N*=3)和定量限(LOQ, *S/N*=10),结果见表2。结果表明,检出限和定量限分别为0.033~0.049 mg/L和0.10~0.15 mg/L。

**表2:** 18种氯代烃类有机溶剂的回归方程、相关系数、检出限和定量限

Compound	Regression equation	*R* ^2^	LOD/(mg/L)	LOQ/(mg/L)
Vinylidene chloride	*Y*=6.57×10^4^*X*-8.87×10^4^	0.9992	0.049	0.15
Dichloromethane	*Y*=2.71×10^4^*X*-5.01×10^4^	0.9994	0.038	0.11
*trans*-1,2-Dichloroethylene	*Y*=6.21×10^4^*X*-8.54×10^5^	0.9998	0.044	0.13
1,1-Dichloroethane	*Y*=7.85×10^4^*X*-1.78×10^6^	0.9992	0.045	0.14
*cis*-1,2-Dichloroethylene	*Y*=5.04×10^4^*X*-1.18×10^6^	0.9995	0.040	0.12
Chloroform	*Y*=4.51×10^4^*X*-8.89×10^5^	0.9993	0.033	0.10
1,1,1-Trichloroethane	*Y*=6.50×10^4^*X*-1.44×10^6^	0.9995	0.046	0.14
Carbon tetrachloride	*Y*=6.05×10^4^*X*-1.69×10^6^	0.9998	0.038	0.11
1,2-Dichloroethane	*Y*=4.45×10^4^*X*-9.63×10^4^	0.9994	0.040	0.12
Trichloroethylene	*Y*=5.11×10^4^*X*-1.33×10^6^	0.9999	0.037	0.11
1,1,2-Trichloroethane	*Y*=2.37×10^4^*X*-4.17×10^5^	0.9994	0.042	0.13
Tetrachloroethylene	*Y*=4.83×10^4^*X*-1.41×10^6^	0.9995	0.047	0.14
1,1,1,2-Tetrachloroethane	*Y*=3.08×10^4^*X*-1.02×10^6^	0.9997	0.039	0.12
1,1,2,2-Tetrachloroethane	*Y*=3.24×10^4^*X*-5.28×10^4^	0.9996	0.041	0.12
1,2,3-Trichloropropane	*Y*=5.42×10^4^*X*-6.13×10^4^	0.9993	0.044	0.13
Pentachloroethane	*Y*=2.19×10^4^*X*-7.24×10^4^	0.9997	0.036	0.11
Hexachloroethane	*Y*=3.93×10^4^*X*-1.60×10^6^	0.9999	0.047	0.14
Hexachloro-1,3-butadiene	*Y*=4.00×10^4^*X*-1.88×10^6^	0.9998	0.048	0.14

*Y*: peak area; *X*: mass concentration, mg/L.

2.5.2 回收率与精密度

在阴性样品(口红(固体)和漱口水(液体))中分别添加3个水平的混合标准溶液,按照1.3节描述进行样品前处理,在优化后的GC-MS实验条件下测定,每个添加水平作6次平行,重复测定两次,计算18种氯代烃类有机溶剂的平均回收率和相对标准偏差(RSD),结果见表3。结果表明,18种氯代烃类有机溶剂在口红和漱口水中的平均回收率为92.4%~103.1%和93.3%~102.4%,相对标准偏差为3.1%~5.3%和2.8%~5.4%,说明该方法准确度好,精密度高,适用于化妆品中目标化合物的测定。

**表3:** 阴性样品中18种氯代烃类有机溶剂的加标回收率和相对标准偏差(*n*=6)

Compound		Added/(mg/kg)		Lipstick						Mouth wash			
				Recovery/%		RSD/%				Recovery/%			RSD/%
Vinylidene chloride		5		92.4		4.3				94.1			5.3
		50		95.7		3.8				96.3			4.3
		500		98.0		4.2				96.2			4.5
Dichloromethane		5		96.3		3.8				95.1			4.0
		50		95.8		4.6				94.9			3.9
		500		97.2		4.0				96.3			4.2
*trans*-1,2-Dichloroethylene		5		98.2		5.0				97.2			4.5
		50		102.3		4.6				96.9			4.6
		500		97.5		4.5				98.4			4.2
1,1-Dichloroethane		5		94.7		4.8				93.5			4.2
		50		96.8		3.2				97.2			4.6
		500		95.8		4.1				96.4			3.7
*cis*-1,2-Dichloroethylene		5		97.3		4.0				95.5			4.6
		50		99.4		4.5				96.1			3.9
		500		98.1		3.7				96.5			4.3
Chloroform		5		94.0		5.1				94.3			4.3
		50		98.3		4.3				95.9			3.5
		500		97.1		4.2				96.7			3.3
1,1,1-Trichloroethane		5		99.2		4.5				98.2			4.9
		50		97.0		4.3				97.9			5.0
		500		96.6		3.9				99.9			4.0
Carbon tetrachloride		5		93.6		4.6				95.1			5.1
		50		96.2		4.5				96.0			4.3
		500		95.3		3.3				95.3			4.6
1,2-Dichloroethane		5		99.3		5.3				98.3			4.3
		50		103.1		4.9				96.2			4.5
		500		98.4		4.5				100.9			3.6
Trichloroethylene		5		95.3		4.3				94.2			4.4
		50		97.2		4.3				97.2			4.2
		500		96.3		3.6				97.3			3.7
1,1,2-Trichloroethane		5		101.2		4.4				93.3			3.8
		50		96.8		4.0				96.4			3.4
		500		99.3		3.1				94.5			2.8
Tetrachloroethylene		5		98.4		3.8				96.2			4.8
		50		98.5		4.2				98.3			5.4
		500		97.2		3.4				97.9			4.6
1,1,1,2-Tetrachloroethane		5		93.9		4.6				102.4			3.9
		50		96.1		4.3				98.4			4.3
		500		95.9		4.7				99.3			4.4
1,1,2,2-Tetrachloroethane		5		94.2		4.0				95.3			5.2
		50		97.3		3.8				98.0			4.2
		500		96.0		3.7				96.5			3.8
1,2,3-Trichloropropane		5		95.1		4.7				94.4			4.6
		50		95.3		5.1				93.7			3.7
		500		97.3		4.3				97.5			4.0
Compound	Added/(mg/kg)		Lipstick						Mouth wash				
			Recovery/%		RSD/%				Recovery/%				RSD/%
Pentachloroethane	5		94.6		4.6				96.3				4.7
	50		98.2		4.7				97.5				5.2
	500		97.4		4.2				98.4				4.6
Hexachloroethane	5		92.7		3.9				97.0				3.7
	50		95.0		4.2				95.0				4.0
	500		94.3		4.0				94.3				3.4
Hexachloro-1,3-butadiene	5		96.8		3.5				98.2				4.9
	50		95.2		3.7				99.5				4.5
	500		98.7		3.2				98.4				4.6

### 2.6 实际样品检测

应用本文建立的方法对市场购买的115个化妆品进行测定,这些化妆品包括口红、香水、指甲油、眼影、粉底液、面霜、乳液、牙膏、漱口水等,涵盖了可能含有氯代烃有机溶剂的大部分商品,具有广泛的代表性。结果表明,3个指甲油样品检测出氯代烃有机溶剂,检出物均为四氯乙烯,不符合《化妆品安全技术规范》中的禁用要求,检出值为11.4~42.0 g/kg。与顶空-GC-MS^[15]^相比,测定结果基本相当,但重复性更佳,且分析时间短。

## 3 结论

本文建立了气相色谱-质谱同时测定化妆品中18种氯代烃类有机溶剂的检测方法。该方法采用高沸点溶剂正十四烷作为提取溶剂,取消溶剂延迟时间,使极易挥发待测物能够得到有效色谱保留和分离,前处理过程利用“盐析”效应,极大程度提高了提取率。采用本方法对115个不同用途的化妆品样品进行测定,结果表明,该方法重复性好,准确度高,灵敏度高,可适用于各种化妆品中氯代烃类有机溶剂的检测,可用于化妆品品质和安全的监控。
